# The *R-CITY* Youth Violence Preventive Intervention: Primary Outcomes from a School Cluster Randomized Controlled Trial

**DOI:** 10.1007/s11121-026-01907-1

**Published:** 2026-04-15

**Authors:** Jessika H. Bottiani, Meredith P. Franco, Michelle K. Francis, Kate Somerville, Chelsea A. Kaihoi, Elise T. Pas, Catherine P. Bradshaw

**Affiliations:** 1 School of Education and Human Development, University of Virginia, PO Box 400281, 405 Emmet Street South, Charlottesville, VA 22904 USA; 2 Johns Hopkins Bloomberg School of Public Health, 415 N. Washington Street, 5th Floor, Baltimore, MD 21231 USA

**Keywords:** Outcomes, RCT, Social and emotional learning, Equity, Classroom

## Abstract

Student experiences of racism and discrimination in schools can undermine their sense of safety and psychological wellbeing and contribute to aggression and violence. Yet educational systems rarely implement violence prevention programming with bias prevention or equity promotion components. To address this gap, researchers and educators partnered to develop *R-CITY* (Reducing Racism and Violence through Collaborative Intervention with Teachers and Youth; Bottiani et al., *School Mental Health, 16*(3), 632–648, 2024). A school-level randomized controlled trial was conducted in 27 elementary and middle schools to assess the ‘value-added’ benefits of supplementing the standard *Second Step* SEL program (22–27 lessons and group implementation support; comparison condition) with *R-CITY*’s equity-focused one-to-one teacher coaching and grade-differentiated sets of six equity lessons with implementation supports (*Second Step* + *R-CITY*, intervention condition). Augmenting *Second Step* with *R-CITY* equity-focused components was associated with significant effects on one of six observational measures of student behavior (physical aggression) and one of three teacher-report measures (general teaching self-efficacy), both in the hypothesized direction. Sensitivity analyses excluding the most severely COVID-impacted cohort identified an additional effect on teacher-reported racial discomfort. No significant effects were found on observed teacher practice outcomes or suspension disproportionality rates. Results provide initial evidence that supplementing traditional SEL programming with equity content and coaching can produce significant incremental effects on select outcomes, including reductions in physical aggression and improvements in teacher capacity; however, further research is needed to evaluate the intervention’s cost-effectiveness and effects on equity-specific outcomes.

**Supplementary Information:**

The online version contains supplementary material available at 10.1007/s11121-026-01907-1.

Experiences of discrimination during adolescence create substantial barriers to psychological wellbeing and academic achievement while increasing engagement in externalizing behaviors associated with violence (Benner et al., 2018). Despite growing evidence of these harmful effects, schools have historically lacked preventive approaches that address violence and discrimination in tandem. The turbulent sociopolitical landscape of the early 2020s, characterized by heightened racial tensions, pandemic-related disruptions, and increasingly polarized debates about equity in schools, underscored the urgent need for integrated approaches to equity and safety. The *R-CITY* (Reducing Racism and Violence through Collaborative Intervention with Teachers and Youth) program was created to supplement an evidence-based social and emotional learning and violence prevention program with equity-focused components (student content and teacher coaching). The current study examined whether *R-CITY* effectively enhanced teacher capacity and practices (e.g., cultural responsiveness), promoted student engagement, and decreased aggression in classrooms. By comparing standard *Second Step* implementation with an enhanced version including additional equity-focused components, this school-level randomized controlled trial provides critical insights into the added value of explicitly addressing equity in youth violence prevention efforts.

## Racism and Discrimination as Risk Factors for Youth Violence

Research shows that societal, institutional, interpersonal, and intrapersonal racism and discrimination in schools are of great public health significance, as they contribute significantly to youth violence and related mental health and adjustment outcomes (Civitillo et al., 2024). Studies have demonstrated robust associations between youth-reported discrimination and subsequent antisocial behavior, externalizing problems, post-traumatic stress, reduced self-esteem, depression, and substance use (Benner et al., 2018). Racially stressful encounters at school may impede youth from having core psychological needs met for competence, autonomy, and belonging, which is essential to school engagement and reduced aggressive behavior. Experiencing microaggressions at school also limits the resilience of African American adolescents exposed to trauma (Woods-Jaeger et al., 2022). Effective interventions must adopt ecological approaches that prevent interpersonal discrimination, foster coping skills for racial stress, and reduce institutional discrimination, such as disproportionate disciplinary practices affecting students of color (Bottiani et al., 2018).

## Preventing Youth Violence by Promoting Social Emotional Learning

Social and emotional learning (SEL) refers to the ways in which young people learn to apply the knowledge, skills and attitudes necessary for healthy identity development, emotional management, goal setting and achievement, empathy development, healthy relationships, and responsible decision making (Collaborative on Academic, Social and Emotional Learning [CASEL], 2026). SEL is often implemented to prevent aggressive behavior and promote youth mental health. Despite meta-analyses demonstrating that SEL programming significantly improves skills, attitudes, behaviors, school climate and safety, peer relationships, school functioning, and academic achievement (e.g., Cipriano et al., 2023), the impacts for minoritized students have been understudied, with research suggesting lack of attention to cultural relevance as well as differential access to and effectiveness of SEL curricula (Cipriano et al., 2024).

In fact, scholars have critiqued traditional SEL curricula, noting that they do not consider social oppression or the experiences of marginalized students, address bias, discrimination, or racism (Camangian & Cariaga, 2022) and instead promote a white, Eurocentric relationship with emotions that is rooted in control and compliance that uphold systems of white supremacy (Lin et al., 2023; Simmons, 2021). Such programming espouses a deficit-based orientation to social and emotional development that centers on “fixing” marginalized youth (DeMartino et al., 2022) and further stigmatizes and marginalizes minoritized students. This is particularly problematic given the connection between negative mental health outcomes for youth of color and reported experiences of racism at school (Centers for Disease Control, 2024).

### Integrating SEL and Equity-Focused Programming

In response, equity-focused SEL approaches have emerged. For example, transformative SEL is a framework that centrally considers equity and justice, specifically focusing on culture, identity, agency, belonging and engagement to promote the development of justice-oriented citizenship (Jagers et al., 2019). CASEL (2026) adopted the concept of transformative SEL, and organizations like the Great Lakes Center have developed tools for the implementation of transformative SEL (Coomer & Skelton, 2019). Transformative SEL studies include youth participatory action research conducted during the COVID-19 pandemic (Owens et al., 2021) and social justice learning communities that strengthen the efficacy of teachers of color to enact equity-based SEL practices (White et al., 2022). However, some have critiqued the current literature for being too conceptual and not practical (DeMartino et al., 2022). Limited emerging outcomes research has a primary focus on high school-aged adolescents (e.g., Pas et al., 2024; Umaña-Taylor et al., 2024).

These critiques highlight a critical gap: upper elementary and middle school students in schools using traditional SEL programs receive limited exposure to content addressing discrimination, bias, or their own racialized experiences, and may encounter frameworks that center dominant cultural norms rather than diverse perspectives. *R-CITY* was developed to fill this gap for grades 4–8 students by providing equity-focused content grounded in transformative SEL principles. Despite the limitations of traditional SEL, wholesale curriculum replacement is often unfeasible for schools given resource constraints, existing investments, and implementation barriers. Rather than expecting schools to replace existing programming, *R-CITY* offers an accessible supplement that introduces transformative SEL components emphasizing culture, identity, and justice-oriented competencies. Co-developed through a research-practice partnership (Bottiani et al., 2024), *R-CITY* features six equity-focused SEL lessons designed to address limitations of existing SEL curricula. The intervention includes equity-focused teacher coaching and implementation supports to help educators effectively facilitate discussions about identity, discrimination, and equity.

## Current Study

This study examined the value-added benefits of the *R-CITY* preventive intervention (i.e., equity-focused set of six lessons and teacher coaching and implementation support supplementing SEL; *R-CITY* SEL + Equity condition) as compared to *Second Step* (Frey et al., 2000) implemented with group support (“SEL Only” condition) in a 27 elementary and middle school randomized trial. A logic model for R-CITY is available in Fig. S2.

Aim 1 examined the proximal effects of the *R-CITY* SEL + Equity condition on teachers' self-reported attitudes and self-efficacy, including self-reported racial discomfort, culturally responsive teaching self-efficacy, and general teaching self-efficacy compared to the SEL Only condition. Aim 2 evaluated impacts on intermediary outcomes such as teachers’ observed classroom practices, including observed culturally responsive practices, and observed effective engagement and emotionally supportive classroom practices. Aim 3 assessed the effects on student outcomes, including observed meaningful participation, disruptive behaviors, and aggressive behaviors. Aim 4 assessed effects on out-of-school suspension (OSS) rates and racial disproportionality in OSS. Taken together, study findings build the research base on impacts of augmenting traditional SEL with equity-focused components for reducing aggression at school.

## Method

### Participants

The sample included a total of 15 elementary and 12 middle schools enrolled in the study as part of four cohorts (one per year) and 145 teachers within these schools.

#### School Demographics

On average, participating schools enrolled 705 students, representing a racially and ethnically diverse population with (in descending order) 47.41% White, 22.39% Black or African American, and 20.06% Hispanic or Latine students. About 46.84% of students qualified for free or reduced-price meals, and the average baseline suspension rate across schools was 10.12% (the average rate at the post timepoint was 14.37%; see Table 1 for school demographics). Baseline equivalence test(s) on the full sample confirmed no significant differences in demographic characteristics between intervention and comparison schools.

**Table 1:** Descriptive statistics

	Intervention Condition			Comparison Condition		
**School Characteristics** (*J* = 27)		*M*	*SD*		*M*	*SD*
Middle School		0.43	0.51		0.46	0.52
Total Enrollment		657.86	222.91		756.23	388.49
Percent Student Population: FARMs Eligible		49.80	19.51		41.48	22.84
Percent Student Population: White		43.15	19.37		51.99	23.40
Teacher Baseline Measures	*n*	*M*	*SD*	*n*	*M*	*SD*
*Classroom Characteristics*						
STEM Subject	68	0.24	0.43	66	0.30	0.46
% Observed Boys	67	48.64	11.59	65	50.97	13.23
% Observed SoC	67	64.53	20.86	65	53.92	27.37
*Teacher Self-Report*						
Years of Teaching Experience	72	8.94	6.72	67	13.46	8.55
Racial Discomfort	70	1.56	0.76	65	1.39	0.79
Culturally-Responsive Teaching Self-Efficacy	70	3.67	0.51	65	3.60	0.48
General Teaching Self-Efficacy	70	3.65	0.76	65	3.71	0.72
*Observer Ratings*						
CARES Practices	68	5.36	2.68	65	6.20	2.52
Anticipation & Responsiveness	67	2.78	0.69	64	2.69	0.72
*Observer Tallies*						
Approvals	62	0.28	0.23	59	0.37	0.33
Disapprovals	56	0.09	0.15	45	0.04	0.07
Reactives	67	0.37	0.24	65	0.43	0.29
**Teacher Demographics** (*N* = 139)						
Grades Taught (*n* = 139)	*n*	%		*n*	%	
4th Grade	23	31.94	-	8	11.94	-
5th Grade	23	31.94	-	21	31.34	-
6th Grade	9	12.50	-	11	16.42	-
7th Grade	7	9.72	-	13	19.40	-
8th Grade	8	11.11	-	10	14.93	-
Mixed Grade	2	2.78	-	4	5.97	-
Sex (*n* = 139)	*n*	%		*n*	%	
Female	57	79.17	-	53	79.10	-
Male	15	20.83	-	12	17.91	-
Prefer not to say	0	0	-	2	2.99	-
Race (*n* = 139)	*n*	%		*n*	%	
Black or African American	9	12.50	-	4	5.97	-
White	54	75.00	-	60	89.55	-
Other	9	12.50	-	3	4.48	-
Ethnicity (*n* = 139)	*n*	%		*n*	%	
Hispanic, Latine, or Spanish Origin	4	5.56	-	1	1.49	-

*FARMs* Free and Reduced-price Meals, *SoC *Students of Color, *M* Mean, *SD* Standard Deviation, *STEM* Science, Technology, Engineering, and Mathematics

#### Teacher Demographics

Most participating teachers self-identified as female (80%), ranging in age from 20 to 50 years (77.2%), and over half (59.0%) reported teaching for nine or more years. Teachers’ self-reported race and ethnicity was White (82.0%), Black or African American (9.4%), Asian or Pacific Islander (2.9%), either Hispanic/Latinx or Native American/American Indian (3.6%), and Others (2.1%). There was a significant difference between the percent of White and non-White teachers *t*(128.01) =  − 2.28, *p* =.024 across conditions, where 75.0% were White in the *R-CITY* SEL + Equity condition and 89.6% were White in the SEL Only condition; this was controlled for in all models. No other significant demographic differences were found between teachers at the intervention and control schools. See Table 1 for baseline demographics and supplemental Fig. S1 for the CONSORT diagram.

#### Coaching Staff Demographics

Throughout the four-year study period, participating schools were supported by six project coaches who brought prior coaching experience and graduate-level exposure to social-emotional learning and equity concepts. All coaches received specialized training in motivational interviewing techniques, the study curricula, and the project’s teacher coaching framework. The coaching team included two licensed, doctoral-level psychologists (clinical or school psychology backgrounds), one licensed clinical social worker, one doctoral-level educational specialist and former teacher, and two master’s-level educational specialists and former certified teachers. The team comprised five women and one man, with coaches self-identifying as African American, White, and mixed race. A project co-investigator with motivational interviewing expertise (Miller & Rollnick, 2002) provided training for all coaching staff.

### Study Design and Procedures

#### Recruitment

The research team recruited four cohorts of schools and teachers over four consecutive years, starting each summer from 2021 to 2025. With the assistance of school district partners, the research team held informational sessions and obtained signed consent from school administrators (e.g., principal), confirming their commitment to participate in the study for the school year. The research team then held teacher recruitment informational sessions and obtained signed consent forms or contact information from interested teachers. A total of 145 teachers submitted signed consent forms prior to baseline data collection and were included in the present analytic sample. The analytic sample included all teachers with at least one data point (self-report, observations), regardless of engagement in intervention activities (i.e., teaching lessons or engaging in training and coaching).

Overall, as shown in the CONSORT figure in the Supplemental Materials, 1121 teachers were assessed for eligibility and ,of these, 599 were not eligible. Of the 522 eligible teachers, 377 declined and 145 consented; thus, the final participation rate was 27.8%. The teacher consent rate varied across cohorts (i.e., 73.7% in Cohort 1 [all elementary], 19.9% in Cohort 2 [mostly middle], 33.3% in Cohort 3 [elementary and middle], and 12.4% in Cohort 4 [all middle]. Our consent rate reflects reasonable engagement given the substantial time commitment for this year-long study and the well-documented post-pandemic challenges of increased teacher workload and research participation reluctance (Carter et al., 2024).

#### Randomized Design, Conditions, and Randomization Procedure

This cluster randomized controlled trial evaluated the effectiveness of an equity-focused social and emotional learning program for reducing discrimination and youth violence (ClinicalTrials.gov NCT05585918). Participating schools were randomly allocated to either a comparison condition (“SEL Only”: *Second Step* implementation with project-provided implementation support) or an intervention condition (“*R-CITY* SEL + Equity”: *Second Step* plus six *R-CITY* equity-focused lessons with enhanced training on equity content and coaching on cultural responsiveness and antibias).

For the first three cohorts, randomization was conducted using the nbpMatching package in R (Beck et al., 2016; R Core Team, 2021) to create optimal pairwise matches based on seven to eight key school covariates (including suspension rates, student economic disadvantage, racial composition, English learner percentages, chronic absenteeism, and test proficiency). Independent *t*-tests confirmed no significant differences across 20 examined covariates, indicating successful balance. The fourth cohort, which included only three schools, required an alternative randomization approach that maintained overall sample balance. Full randomization procedures and covariate details are provided in the supplemental online materials.

#### Student Curricula

##### Second Step

All schools implemented the *Second Step* (Frey et al., 2000) SEL curriculum; it included 22–27 lessons for grades 4–8, focused on the core SEL models in the CASEL framework. The curriculum was delivered electronically for middle school and available in both electronic and print formats for elementary; both included implementation resources.

##### ***R-CITY*** Equity Supplement

The *R-CITY* intervention condition augmented the *Second Step* curriculum with novel teacher-delivered and facilitated equity lessons for students and provided teacher implementation supports designed to enhance teachers’ implementation of the equity content specifically. The *R-CITY* equity lessons and lesson plans were co-developed with the partner district (see Bottiani et al., 2024) and followed a similar instructional format and sequencing as *Second Step* to ease implementation burden for teachers, while maintaining distinct *R-CITY* branding and design. The student content consisted of six, 30-min teacher-led, equity-focused SEL lessons with three grade-level versions (grades 4–5, 6, and 7–8), delivered weekly during morning advisory periods. Topics included identity and belonging, personal agency, histories of injustice, bias awareness, and equity action planning, while also emphasizing self-care and self-compassion in response to experiencing bias. Each lesson followed a consistent four-component structure: a Warm-Up for engagement and review, an Introduction of new concepts, a Discussion and Activity, and a Wrap-Up to assess understanding. All lesson materials were accessible to teachers through a Google Drive. Combined with *Second Step*’s 22–27 lessons (grades 4–8), the intervention condition totaled 28–33 lessons depending on grade. Teachers in the *R-CITY* SEL + Equity condition also received a 90-min group training session, coordinated with school’s equity lead, providing an overview of the content within the six equity-focused student lessons.

#### Teacher Coaching Models

##### Standard Implementation Coaching (Comparison Condition—SEL Only)

SEL Only teachers received district-provided training and structured implementation support from project staff for *Second Step*. Following the initial engagement of school leadership, coaches conducted a *Second Step* Implementation Needs Assessment Interview to gather detailed information about teachers’ experiences implementing *Second Step* and present a menu of optional individualized supports, such as classroom observations with feedback, resource sharing, or consultative meetings to enhance their implementation skills. These were summarized for school leadership to identify needs and co-plan group professional development session that was subsequently implemented by coaches. Coaching was guided by motivational interviewing principles and maintained non-evaluative teacher-coach relationships (Miller & Rollnick, 2002, 2023).

##### Enhanced Equity-Focused Coaching (Intervention Condition – ***R-CITY*** SEL + Equity)

The *R-CITY* SEL + Equity intervention condition included individual teacher coaching. The *R-CITY* coaches engaged in an equity-focused adaptation of the evidence-based Classroom Check-Up (CCU; Reinke et al., 2011) coaching model to foster equity-centered classroom practices and mitigate discriminatory and racist classroom dynamics (see Bottiani et al., 2024; Franco et al., 2025). Like the SEL Only condition, coaching was guided by motivational interviewing and non-evaluative relationships but provided more intensive 1-on-1 support. This enhanced coaching approach included individualized support to help teachers implement culturally sustaining practices while building “equity stamina”, which was the partner district’s term for building equity-informed teaching strengths through consistent practice. Coaching following a five-step process: (1) reconnecting teachers with core values, (2) eliciting teacher-identified change targets, (3) mapping evidence-based strategies to goals, (4) creating implementation plans, and (5) providing classroom observation feedback (Franco et al., 2025). Coaching delivery evolved across cohorts due to COVID-19. Cohort 1 (2021–2022) was primarily virtual, with predominantly in-person delivery beginning in Cohort 2 (2022–2023) and continuing through Cohort 4 (2024–2025); Cohort 3 comparison coaching included some virtual sessions due to staffing constraints while intervention coaching remained in-person.

#### Implementation Overview and Data

The intervention condition coaches supported implementation of both *Second Step* and the equity-focused lessons, though teachers prioritized equity content during sessions consistent with the motivational interviewing approach that honors teacher autonomy (Franco et al., 2025).

##### Second Step Implementation Data

Implementation data were obtained through the *Second Step* digital learning management system, which provided automated usage analytics through their online portal. Across conditions, teachers’ digital implementation metrics indicated that 54 of 123 teachers with *Second Step* dashboard data (43.9%) met implementation criteria by delivering at least two-thirds of the total number of *Second Step* lessons for their grade level. The 22 teachers missing *Second Step* data did not have an online profile and therefore were not included in school district administrative reports on *Second Step* implementation. On average, digital implementation metrics indicated that participating teachers assigned to the SEL Only condition completed 54% of *Second Step* lessons assigned to their grade, though this varied by cohort (C1: 97%, C2: 37%, C3: 64%, C4: 18%). By comparison, digital implementation metrics indicated that participating teachers assigned to the Intervention condition completed 51% of *Second Step* lessons assigned to their grade, on average. This also varied by cohort (C1: 80%, C2: 28%, C3: 54%, C4: 41%). See supplemental Table S1 for more information.

##### Equity Curriculum Implementation Data

Implementation data for the equity curriculum were collected through teacher self-reports and coach observations of lesson delivery. Among the 73 participating teachers in the *R-CITY* SEL + Equity condition, 48 teachers (65.8%) met the implementation criteria of delivering at least four of the six lessons (66.7% of curriculum components). However, there were significant differences in equity lesson implementation across cohorts. Specifically, 0% of Cohort 1 teachers (*n* = 0), 63% of Cohort 2 teachers (*n* = 12), 95% of Cohort 3 teachers (*n* = 21), and 89% of Cohort 4 teachers (*n* = 8) implemented at least four lessons. Teachers were asked to rate students’ and their own comfort level on a 5-point scale (1-Very Uncomfortable to 5-Very Comfortable). Data indicated that students were perceived to be comfortable with *R-CITY* lesson content (*M* = 3.9, *SD* =.75) and comfortable with others during *R-CITY* lesson delivery (*M* = 4.0, *SD* =.83 for comfort with peers and *M* = 4.3, *SD* =.65 for comfort with the teacher). Teachers also reported high comfort with teaching the *R-CITY* lessons (*M* = 4.57, *SD* =.58). Lesson preparation time was low on average (*M* = 12.49 min, *SD* = 8.23) though varied widely (5.00–41.25 min).

##### Standard Coaching (SEL Only Condition) Dosage Data

Coaches documented an average of 3.22 contacts with 51 teachers (76% of all 67 teachers in the condition) who received coaching in the SEL-Only condition. Each coaching case averaged 53.43 min of direct teacher interaction. Coach ratings indicated positive teacher engagement and minimal resistance.

##### Enhanced Equity-Focused Coaching (***R-CITY*** SEL + Equity Condition) Dosage Data

Relative to the SEL-Only condition, *R-CITY* SEL + Equity coaches provided more intensive one-on-one support designed to facilitate teachers’ implementation of six equity-focused SEL lessons. Coaches documented an average of 6.11 contacts with the 55 teachers (85.9% of the total 64 teachers in this condition who spent at least 15 min with the coach; see Franco et al., 2025). Each coaching case averaged 186.27 min (approximately 3.1 h) of direct teacher interaction. Furthermore, coach-ratings in their coaching contact logs revealed consistently positive scores on teacher affect and engagement of teachers and minimal instances of discomfort or resistance in the *R-CITY* condition (see Franco et al., 2025).

The higher coaching dosage in the intervention condition represents a core feature of the intervention model (i.e., an intended increased intensity) and not an experimental confound. Although both conditions utilized a four-step coaching protocol, the *R-CITY* enhanced coaching incorporated more content and substantive focus (i.e., integration of equity concepts into their SEL instruction) executed through additional coaching contacts. This dosage differential aligns with literature suggesting that transformative pedagogical changes require more intensive rather than incremental professional learning (Darling-Hammond et al., 2017).

### Measures and Data Collection Procedures

Data were collected at baseline (fall) and post-intervention (spring). Table 1 provides baseline measures of outcomes for intervention and comparison conditions.

#### Teacher Self-Report Survey

Teachers completed an online survey at both timepoints assessing various aspects of their attitudes and efficacy. Items in the teacher self-report were selected to represent key facets of each construct while minimizing redundancy. The Racial Discomfort Scale (Knowles & Hawkman, 2020) utilized seven items (*α* =.82) to evaluate teachers’ comfort discussing issues pertaining to race and racism. The Culturally Responsive Teaching Self-Efficacy Scale (Siwatu, 2007) employed 15 items (*α* =.80) to assess teachers’ perceived ability to implement culturally responsive practices and meaningfully connect with students from diverse backgrounds. Additional measures included general teacher self-efficacy (Hoy & Woolfolk, 1993; three items; *α* =.67), social desirability (Strahan & Gerbasi, 1972; five items; *α* =.63), and demographic information. Due to the comprehensive nature of our assessment battery, we used abbreviated versions of several validated scales to reduce respondent burden while maintaining construct coverage. Specifically, we used 7 of 18 items from the Racial Discomfort Scale (Knowles & Hawkman, 2020), 15 of 40 items from the Culturally Responsive Teaching Self-Efficacy Scale (Siwatu, 2007), 3 of 5 items from the General Teacher Self-Efficacy scale (Hoy & Woolfolk, 1993), and 5 of 10 items from the Social Desirability scale (Strahan & Gerbasi, 1972). Items were selected to represent key facets of each construct while minimizing redundancy. Internal consistency estimates for abbreviated scales ranged from α =.60–.81, with lower estimates in the.60–.69 range best characterized as modest., Their use should be interpreted with appropriate caution.

#### Observed Classroom Teacher Practices and Student Behaviors

##### Assessing School Settings: Interactions of Students and Teachers (ASSIST)

Independent, trained data collectors blind to intervention status conducted classroom observations using the *Assessing School Settings: Interactions of Students and Teachers* (ASSIST), a validated observational tool (Bradshaw et al., 2024; Rusby et al., 2011). The protocol included two 18-min observations per teacher. Observers first documented classroom context (approximately 3 min), then recorded event-based tallies for 15 min, focusing on teacher proactive behavior expectations, approvals, opportunities to respond, disapprovals, and reactive behavior management (hereafter referred to as “reactives”) as well as student disruptive, non-cooperative, and aggressive behaviors. Observer trainees were certified as reliable in the ASSIST after passing the in-school reliability assessment with an 80% or higher threshold for inter-rater reliability with expert observers across student and teacher tallies. Initial training inter-rater reliability for Cohorts 1–4 observations on the tallies averaged 85.2%, with 89.8% reliability during mid-study recalibration. All tallies were normalized by dividing the total number of practices or behaviors for each tally by the duration of the observation in minutes. Following tallying, observers completed global ratings on several subscales: teacher anticipation and responsiveness (six items; *α* =.82), meaningful participation (four items; *α* =.81), student cooperation (four items; *α* =.93), and student socially disruptive behavior (four items; *α* =.76). These utilized 5-point scales from 0 (never) to 4 (almost continuously), except for student disruptive behaviors which ranged from 0 (never occurred) to 4 (often occurred, ~ 6 + times).

##### CARES Culturally Responsive Practices Observational Measure

The CARES Observational Measure (Franco et al., 2024) is an observational assessment tool used to conduct structured classroom observations of teachers’ culturally responsive practices (CRPs). Each item is scored on a scale including (−) 1 (*needs improvement*), 0 (*not observed*), and (+) 1 (*well done*) based on their observations during the observation period. Importantly, *needs improvement* was operationalized as potentially harmful implementation of the practice rather than simply poor execution, distinguishing it from practices that were *not observed* during the observation period. Observer trainees were certified as reliable in the CARES measure after passing the video-based reliability assessment with a 75% or higher threshold for inter-rater reliability with expert observers across all items. Initial training inter-rater reliability for Cohorts 1–4 observations averaged 82.1%, with 79.4% reliability during mid-study recalibration. In the current study, the CARES scale demonstrated moderate internal consistency; McDonald’s omega reliability statistic was 0.72 for the 19-item measure, on average across all cohorts and time points. We calculated McDonald’s omega, consistent with the original measure validation paper (Franco et al., 2024), as it is favored over Cronbach’s alpha when measures are newly developed or still in progress (Hayes & Coutts, 2020).

##### Observation Sequencing and Selection

Both observational measures were administered in their complete, validated form without modification. The combined observation protocol was to administer the CARES (30-min period) before the ASSIST observation (~ 20-min period). For all observational analyses, morning observations served as the focal observation when multiple observations per teacher were available, as the advisory period was in the morning and this approach minimized confounding from daily disruptions (e.g., schedule changes, behavioral incidents) more common in afternoon periods.

#### School-Level Data

The state department of education publicly released data that were used for this study. Analytic covariates included out-of-school suspension rates (total, and disaggregated by race), student enrollment, racial/ethnic composition of student population, and percentage of students qualifying for free and reduced-price meals (FARMs). Note that suspension data at baseline was considered the school year prior to intervention implementation, and suspension data at post was considered the school year of intervention implementation.

### Data Analysis

Intent-to-treat (ITT) analyses were conducted. All teachers for whom outcome data were available at post-intervention, consistent with contemporary standards for cluster randomized controlled trials (Gupta, 2011; Hollis & Campbell, 1999), were included in analyses regardless of their level of participation in the intervention activities. This approach preserves the benefits of randomization by maintaining balanced groups and provides a more realistic estimate of intervention effectiveness under real-world implementation conditions. To examine intervention effects, we conducted a primary analysis using data from all four cohorts combined. Given that Cohort 1 implementation occurred during the first year of the COVID-19 pandemic when classrooms frequently transitioned between in-person, virtual, and hybrid learning formats, we conducted a sensitivity analysis using only data from Cohorts 2–4 to examine intervention effects under more stable implementation conditions. This approach assessed whether the pandemic-related disruptions in Cohort 1 might have influenced the overall intervention effects.

Multilevel linear regression models were used to account for the nested structure of the data, with teachers (and their classrooms) nested within schools. Separate multilevel models were conducted for each outcome, with school as a random effect. All multilevel models were specified in the following form:

$${Y}_{ij}={\gamma }_{00}+{\gamma }_{01}{\left(Intervention\right)}_{j}+{\gamma }_{10}{\left(Covariates\right)}_{ij}+{\gamma }_{0k}{\left(School-level\;Covariates\right)}_{j}+{u}_{0j}+{r}_{ij}$$

where ${Y}_{ij}$ represents the outcome for teacher i in school j, ${\gamma }_{00}$ represents the intercept, ${\gamma }_{01}$ represents the intervention effect, ${\gamma }_{10}$ represents the effects of teacher-level covariates, ${\gamma }_{0k}$ represents the effects of school-level covariates, ${u}_{0j}$ represents the random effect for school j, and ${r}_{ij}$ represents the residual error.

In addition, we ran school-level only regression model estimates for disproportionality of discipline in the classroom across three metrics: base rate of Black student suspensions, relative risk ratio, and the raw differential representation metric. Black student’s suspension rate was calculated as the number of Black students suspended divided by the number of Black students enrolled. A rate ratio was created by dividing Black students’ suspension rate by White students’ suspension rate. When the comparison group had 0% risk, we added 1 percentage point to both groups’ risk calculations to prevent undefined risk ratios while maintaining analytical consistency, an approach similar to continuity corrections used in epidemiological research (Sweeting et al., 2004). Finally, Raw Differential Representation (RDR; Girvan et al., 2019) was calculated by determining the risk difference between groups (focal group suspension rate minus comparison group rate) and multiplying by the focal group’s total enrollment to estimate the number of excess students suspended attributable to disproportionality. The RDR estimates the number of students in a specific group who were suspended but would not have been under equitable discipline circumstances (i.e., had their rate of discipline been the same as students in the reference group), providing a concrete indicator of how many students are impacted by disproportionate disciplinary practices.

Standardized regression coefficients (β) and 95% confidence intervals (CIs) are reported for all analyses. To control for multiple comparisons within each outcome domain, we applied the Benjamini-Hochberg (1995) procedure to adjust *p*-values separately for student behavior outcomes and teacher practice outcomes. Results were considered statistically significant if their Benjamini–Hochberg adjusted *p*-values were less than.05. Teacher report and suspension-related outcomes were analyzed as secondary exploratory measures, consistent with the clinical trials registry, and thus a correction was not applied to these outcomes.

### Covariates

Models included: teacher race/ethnicity (1 = teacher of color), sex (1 = woman), grade level taught, years of teaching experience, subject area (1 = STEM vs. 0 = non-STEM), social desirability bias (for teacher self-reported outcomes), and the demographic composition of observed students (% students of color, % boys, for the observed outcomes), along with baseline outcome measures. School-level covariates included implementation cohort (Cohorts 2–4 vs. Cohort 1), student body demographics (% students of color, % eligible for FARMS), and school enrollment size to control for organizational and community characteristics that might influence intervention effects.

### Missing Data Analysis

Teacher self-reported outcomes were missing for 20–24 teachers at post-survey (different *n* depending on measure, roughly 14%–17%). A greater proportion of intervention group teachers (20–24%), in contrast to 9% of comparison group teachers, did not respond to the post-test survey (or portions of the survey). Observer reports of outcomes were missing for 19–20 teachers at post-test depending on the measure (ranging from 10 to 21%). A greater proportion of intervention group teachers (~ 21%) were not observed at post (depending on the measure) relative to teachers in the comparison group (10%–11%). However, none of the *t-*tests for these comparisons were statistically significant. Missingness on post was not significantly correlated with baseline scores on teacher self-report or observer reports. Supplemental analyses (Table S2) revealed no significant baseline differences between completers (pre and post) and non-completers (pre-test only), suggesting attrition was not systematically related to observable teacher factors, including race/ethnicity (16% vs. 14% teachers of color), sex (79% vs. 86% women), subject area (27% vs. 33% STEM), school level (46% vs. 43% middle school), or years of teaching experience (*M* = 11.30 vs. 10.07 years; all *p*s >.40). Therefore, FIML was used.

## Results

All results presented below applied Benjamini-Hochberg (1995) false discovery rate correction separately within student behavior and teacher practice outcome domains to account for multiple comparisons. To provide transparency about the robustness of findings, tables present both adjusted and unadjusted *p*-values for intervention effects. Where findings were statistically significant before correction but did not survive adjustment, we note this in tables to aid interpretation and support future meta-analyses. In the narrative results below, we discuss only effects that remained significant after adjustment.

### Aim 1: Effects on Teachers’ Self-Reported Racial Attitudes and Teaching Efficacy

Table 2 presents the estimated intervention effects on teacher self-reported attitudes and self-efficacy. Teachers in the *R-CITY* SEL + Equity condition reported significantly higher levels of General Teaching Self-Efficacy (*β* = 0.20, 95% CI [0.08, 0.32]) compared to teachers in the SEL-Only condition. Teachers in the *R-CITY* SEL + Equity condition had lower Racial Discomfort and higher Culturally Responsive Self-Efficacy than SEL Only teachers, but these differences were not significant. Sensitivity analyses focused on Cohorts 2–4 only indicated a significant effect on Racial Discomfort, with *R-CITY* SEL + Equity teachers having lower Racial Discomfort than SEL Only teachers (*β* =  − 0.27, 95% CI [− 0.44, − 0.10]). *R-CITY* SEL + Equity teachers also reported significantly higher General Teaching Self-Efficacy (*β* = 0.30, 95% CI [0.18, 0.43]) compared to SEL Only teachers in the C2-C4 sensitivity analyses. The effect on Culturally Responsive Self-Efficacy remained non-significant (*β* = 0.15, 95% CI [− 0.05, 0.35]).

**Table 2:** Multilevel linear regression model estimates for self-reported and observed teacher self-efficacy, attitudes, and practices

	*Teacher Self-Report*						*Classroom Observations of Teacher Practices*									
							*Observer Ratings*				*Observer Tallies*					
	General Teaching Self-Efficacy		Culturally Responsive Self-Efficacy		Racial Discomfort		CARES Practices		Anticipation & Responsiveness		Approvals		Disapprovals		Reactives	
	*N* = 139		*N* = 139		*N* = 139		*N* = 115		*N* = 107		*N* = 107		*N* = 107		*N* = 107	
	*β*	CIs	*β*	CIs	*β*	CIs	*β*	CIs	*β*	CIs	*β*	CIs	*β*	CIs	*β*	CIs
**Teacher-level**																
Teacher of Color	.019	−.14,.18	.018	−.15,.18	−.001	−.23,.22	−.077	−.26,.11	−.054	−.26,.15	−.005	−.17,.16	−.021	−.29,.25	−.021	−.24,.20
Woman	.069	−.09,.23	−.026	−.16,.11	−.069	−.25,.11	.119	−.00,.24	.151	−.10,.31	.067	−.13,.27	.009	−.20,.22	.082	−.06,.22
Grade Taught	.041	−.16,.24	.082	−.12,.29	−.187	−.41,.03	.009	−.32,.34	.044	−.37,.46	−.198	−.42,.02	−.014	−.52,.50	−.394	−.68,-.11
Years Teaching	.208	.08,.34	.067	−.09,.22	−.124	−.25,-.001	−.084	−.27,.10	.062	−.14,.26	.016	−.13,.16	.069	−.20,33	.063	−.15,.27
STEM Subject	−.076	−.29,.14	−.212	−.35,-.08	.296	.18,.41	.014	−.20,.23	−.008	−.26,.25	−.009	−.36,.34	.395	.09,.70	.261	.02,.50
SD Bias	.051	−.12,.22	−.038	−.23,.16	.057	−.11,.22	-	-	-	-	-	-	-	-	-	-
% Observed SoC	-	-	-	-	-	-	.067	−.21,34	.063	−.25,.37	−.002	−.27,.27	.110	−.14,.36	−.050	−.31,.21
% Observed Boys	-	-	-	-	-	-	-	-	.048	−.16,.25	−.140	-.28,.004	.109	−.12,34	.206	.05,.36
Baseline Outcome	.563	.43,.70	.568	.43,.71	.518	.31,.73	.106	−.15,.36	.237	.02,46	.186	−.01,.38	.133	−.13,.40	.155	−.01,.32
**School-level**																
R-CITY (C1-C4)	.199**	.08,32	.052	−.11,.21	−.137	−.30,.03	−.139	−.31,.03	.065	−.17,.30	−.107	−.26,.05	.071	−.15,.30	−.006	−.23,.22
R-CITY (C2-C4)	.303**	.18,.43	.149	−.05,.35	−.268**	−.44,-.10	−.135	−.33,.06	.185	−.03,.40	−.224†	−.44,-.01	.228†	.01,.45	.070	−.18,.32
Cohort 2	−.052	−.27,.17	−.001	−.21,.21	.057	−.13,.24	−.233	−.56,.10	−.004	−.53,.53	.162	−.15,.47	−.232	−.62,.15	.003	−.40,.41
Cohort 3	−.105	−.27,.06	−.082	−.28,.11	.107	−.05,.26	−.151	−.38,.08	−.015	−.38,.35	−.057	−.29,.18	.123	−.12,.37	.261	.06,.46
Cohort 4	.014	−.15,.18	.016	−.15,.18	.107	−.04,.26	.135	−.11,.38	−.025	−.36,.31	.088	−.12,.30	.083	−.19,.35	.161	−.05,.37
% Students of Color	−.057	−.29,.18	−.067	−.33,.19	.035	−.17,.24	.039	−.53,.61	−.087	−.90,.73	.004	−.42,.42	−.149	−.67,.37	.505	.12,.88
% FARMS Eligible	.018	−.20,.24	.006	−.29,.30	.051	−.20,.31	−.208	−.74,.32	.029	−.69,.75	−.096	−.50,.31	.152	−.42,.73	−.324	−.73,.08
Enrollment Size	−.158	−.40,.08	−.112	−.34,.11	−.024	−.26,.21	−.028	−.39,.33	−.109	−.56,.34	.148	−.25,.54	−.017	−.47,.44	.089	−.25,.43

*β* = standardized regression coefficients. CIs = 95% confidence intervals. % FARMS Eligible means the percent of the total student enrollment of the school eligible for free and reduced-priced meals. Reference groups for dummy coded covariates are as follows: Race (White); Sex (Male); Subject (Non-STEM or STEM-non-STEM combined). *SD Bias*, Social desirability bias. SD Bias omitted from models with observed outcomes. % Observed Male means the percent of the total number of students in the classroom with a gender identity expression observers perceived to be male. % Observed SoC means the percent of the total number of students in the classroom that observers perceived phenotypically to be students of color. % Students of Color means the percent of the total student enrollment of a school who were students of color. Models were run separately for each outcome, covariate *β*s and CIs reported here for the C1-C4 models only. Sensitivity analyses were conducted by removing Cohort 1 from the dataset (see row: R-CITY C2-C4). Due to some variation in ASSIST observation time (range 10.6–18 min), tallies were normalized per minute. *P*-values are adjusted using Benjamini–Hochberg correction for the classroom observations of teacher practices. */** *p* <.05/.01 (B-H adjusted); †/†† *p* <.05/.01 (unadjusted). Significance markers are shown only for intervention effects. For covariates, statistical significance can be inferred from confidence intervals that exclude zero

### Aim 2: Effects on Teachers’ Observed Classroom Practices

The analyses found no significant differences between conditions on observed teacher practices (Table 2). Effects on CARES Practices, Anticipation & Responsiveness, Approvals, Disapprovals, and Reactives were all non-significant. In sensitivity analyses focused on Cohorts 2–4, effects remained non-significant.

### Aim 3: Effects on Student Aggression and Behavior

Table 3 presents the intervention effects on observed student behaviors. Students in the *R-CITY* SEL + Equity condition showed significantly lower Physical Aggression (*β* =  − 0.31, 95% CI [− 0.51, − 0.11]) compared to students in the SEL Only condition. The effects on other student behavior measures (Meaningful Participation, Student Cooperation, Socially Disruptive Behavior, Disruptives, and Verbal Aggression) were non-significant. In sensitivity analyses focused on Cohorts 2–4, effects were non-significant.

**Table 3:** Multilevel linear regression model estimates for observed student behaviors in the classroom

*Classroom Observations of Students*												
	*Observer Ratings*								*Observer Tallies*			
	Meaningful Participation		Cooperation		Socially Disruptive Behavior		Disruptives		Verbal Aggression		Physical Aggression	
	*N* = 106		*N* = 107		*N* = 107		*N* = 107		*N* = 107		*N* = 107	
	*β*	CIs	*β*	CIs	*β*	CIs	*Β*	CIs	*β*	CIs	*β*	CIs
**Teacher-level**												
Teacher of Color	.023	−.23,.27	−.071	−.24,.09	−.059	− 23,.12	−.025	−.17,.12	−.166	−.31, −.02	−.134	−.34,.07
Woman	−.136	−.28,.01	−.075	−.25,.10	.037	−.10,.18	.035	−.04,.11	.040	−.12,.20	.019	−.12,.16
Grade Taught	−.141	−.43,.15	.060	−.23,.35	.213	−.07,.50	.347	−.10,.79	−.020	−.32,.28	−.063	−.30,.18
Years Teaching	.052	−.14,.25	.119	−.06,.30	−.167	−.37,.04	−.012	−.27,.24	−.109	−.26,.04	−.268	−.43, −.11
STEM Subject	.175	−.10,.45	−.112	−.30,.08	.068	−.23,.36	−.137	−.33,.06	.159	.06,.26	.074	−.21,.36
% Observed SoC	.19	−.11,.50	.025	−.21,.26	−.035	−.28,.21	.031	−.14,.20	.200	.08,.32	−.029	−.24,.19
% Observed Boys	−.140	−.31,.03	−.106	−.28,.07	.052	−.09,.19	.082	−.14,.30	−.262	−.63,.11	−.232	−.44, −.02
Baseline Outcome	.065	−.14,27	.286	.05,.52	.228	−.02,.48	.353	.07,.64	−.092	−.20,.02	−.090	−.28,.10
**School-level**												
R-CITY (C1-C4)	−.104	−.25,.05	.049	−.15,.25	−.168	−.36,.03	−.084	−.30,13	−.159†	− 30,. −.02	−.311*	−.51, −.11
R-CITY (C2-C4)	−.085	−.24,.07	.058	−.14,26	−.183	−.42,.06	−.095	−.33,.14	−.182†	−.36, −.002	−.371†	−.65, −.09
Cohort 2	−.070	−.46,.32	−.322	−.73,.09	.305	−.06,.67	.113	−.16,.39	.128	−.15,.40	.180	−.05,.41
Cohort 3	.172	−.08,.42	−.177	−.40,.05	.292	.02,.57	.338	.15,.52	.083	−.11,.28	.128	−.05,30
Cohort 4	.087	−.17,.34	−.175	−.36,.01	.174	−.04,.39	.239	.01,.47	.135	−.01,.28	.159	.03,.35
% Students of Color	.021	−.57,.61	.034	−.64,.70	−.150	−.94,.64	−.303	−.83,.23	−.176	−.65,.30	.200	−.37,.77
% FARMS Eligible	−.348	−.92,.22	−.080	−.70,.54	.109	−.63,.85	.287	−.17,.74	.187	−.28,.65	.126	−.46,.71
Enrollment Size	−.175	−.58,.23	−.133	−.45,.18	−.033	−.34,.27	−.162	−.52,.20	.059	−.28,.40	.172	−.12,.47

*β* = standardized regression coefficients. CIs = 95% confidence intervals. % FARMS Eligible means the percent of the total student enrollment of the school eligible for free and reduced-priced meals. Reference groups for dummy coded covariates are as follows: Race (White); Sex (Male); Subject (Non-STEM or STEM-non-STEM combined). % Observed Male means the percent of the total number of students in the classroom with a gender identity expression observers perceived to be male. % Observed SoC means the percent of the total number of students in the classroom that observers perceived phenotypically to be students of color. % Students of Color means the percent of the total student enrollment of a school who were students of color. Models were run separately for each outcome, covariate *β*s and CIs reported here for the C1-C4 models only. Sensitivity analyses were conducted by removing Cohort 1 from the dataset (see row: R-CITY C2-C4). Due to some variation in ASSIST observation time (range 10.55–18 min), tallies were normalized per minute. *P*-values are adjusted using Benjamini–Hochberg correction for observations of student behavior. */** *p* <.05/.01 (B-H adjusted); †/†† *p* <.05/.01 (unadjusted). Significance markers are shown only for intervention effects. For covariates, statistical significance can be inferred from confidence intervals that exclude zero

### Aim 4: Effects on Out-of-School Suspensions and Discipline Disproportionality

Table 4 presents the intervention effects on observed OSS and racial disproportionality in OSS. Schools in the *R-CITY* SEL + Equity condition did not show significant effects on disproportionality measures.

**Table 4:** Regression model estimates for disproportionality of discipline in the classroom

	*Disproportionality Metrics*					
	Base Rate of Black Student Suspensions		Relative Risk Ratio		Raw Differential Representation	
	J = 27		J = 27		J = 27	
	*β*	CIs	*β*	CIs	*β*	CIs
**School-level**						
R-CITY (C1-C4)	−.037	−.20,.13	.084	−.19,.35	−.200	−.44,.04
% Students of Color	.304	−.04,.65	.355	−.16,.87	.406	−.02,.83
% FARMS Eligible	.020	−.37,.41	−.518	− 1.04,.001	−.170	−.64,.30
Enrollment Size	−.215	−.52,.09	−.121	−.60,.36	.053	−.40,.51
Middle School	.937	.63,1.25	.170	−.29,.63	.449	−.04,.94
Disproportionality Metric (Baseline)	−.029	−.28,.22	.626	.43,.82	.181	−.14,.50

*β* = standardized regression coefficients. CIs = 95% confidence intervals. The reference group for the Middle School covariate was “Elementary School”. % FARMS Eligible means the percent of the total student enrollment of the school eligible for free and reduced-priced meals. % Students of Color means the percent of the total student enrollment of a school who were students of color

## Discussion

Multilevel analyses comparing *R-CITY* SEL + Equity to an active control condition (traditional SEL only) revealed significant effects on General Teaching Self-Efficacy and student Physical Aggression across all cohorts, with an additional effect on teacher Racial Discomfort emerging in Cohorts 2–4 sensitivity analysis to account for when implementation was more fully realized without the extensive COVID-related interruptions (Harmey & Moss, 2023) impacting Cohort 1. These effects demonstrate that targeted equity supplements can provide value beyond traditional, otherwise comprehensive SEL programming. The findings suggest that equity-focused supplements can shift teacher mindsets and reduce certain forms of student aggression, though achieving observable changes in teaching practices may require more intensive supports, extended implementation and/or measurement time, or alternative models such as transformational SEL with equity fully integrated rather than supplemented. The detection of effects despite the active comparison condition receiving comprehensive SEL programming (22–27 lessons) demonstrates the meaningful yet incremental value of the equity supplement approach. However, the absence of effects on observed teacher cultural responsiveness and other practices limits understanding of the intervention's mechanisms of action. The difference between results for all cohorts versus Cohorts 2–4 underscores how implementation quality, shaped by both pandemic-related disruptions and sociopolitical tensions surrounding equity initiatives, influenced effectiveness.

### Effects on Teacher Mindsets and Practices

Teachers in the *R-CITY* SEL + Equity condition reported significantly higher General Teaching Self-Efficacy compared to teachers in the SEL Only condition. Improved efficacy could have stemmed from the experience of success in implementing the *R-CITY* SEL + Equity content, the clarity and support surrounding the discussion of difficult topics in this condition, and/or experiences with that the intensive one-on-one coaching provided in the *R-CITY* SEL + Equity condition. This aligns with existing research that similarly shows the impacts of coaching on teacher efficacy (e.g., Walsh et al., 2020). In sensitivity analyses for Cohorts 2–4, the General Teaching Self-Efficacy finding persisted and was slightly stronger. This content may have been particularly salient during a time when many teachers reported feeling uncertain about whether or how to discuss issues of bias and discrimination in class (Woo et al., 2024).

Although no effects were found for Racial Discomfort in the main analyses, in sensitivity analyses focused on Cohorts 2–4 with fuller coaching implementation, *R-CITY* SEL + Equity teachers reported significantly reduced Racial Discomfort, indicating that teachers in the intervention condition felt more comfortable confronting issues of race and racism in the context of greater fidelity. This is likely related to the subject matter of the *R-CITY* SEL + Equity lessons; exposure to delivering lessons on racial bias and discrimination to their classes likely made teachers more comfortable with issues of race and racism. Overall, these patterns of stronger and additional effects in Cohorts 2–4 on teacher capacity suggest potential COVID-19 pandemic-related implementation differences possibly influenced the intervention's effectiveness.

When considering literature on how teachers’ hesitancy to address race can affect the well-being of students, and particularly Black students (Henderson et al., 2019), the reduction in teacher Racial Discomfort found in Cohorts 2–4 represents a meaningful shift in teacher mindset that may support more effective cross-racial interactions in classrooms. However, this attitudinal change did not translate to observable improvements in teaching practices as measured by the ASSIST or the CARES, suggesting that reduced Racial Discomfort alone may be insufficient to produce practice change. In particular, the non-significant findings for both self-reported Culturally Responsive Teaching Self-Efficacy and CARES observed culturally responsive practices suggest that the intervention’s equity components, as currently designed and implemented, did not achieve the hypothesized improvements in cultural responsiveness.

Future research should examine whether more intensive coaching supports, extended implementation periods, or comprehensive transformational SEL approaches are needed to bridge the gap between shifts in teacher comfort and actual changes in classroom practice. Additionally, the measurement of culturally responsive practices may not fully capture the specific equity-focused behaviors targeted by *R-CITY*. While culturally responsive pedagogy emphasizes responsiveness to cultural differences, it may be less attuned to the explicit bias reduction and discrimination prevention practices that were central to the intervention's equity focus. Future work should develop or adapt observational measures that specifically assess anti-bias teaching practices and justice-oriented classroom interactions. Our team is currently developing an enhanced version of the CARES observed culturally responsive practices measure that explicitly incorporates anti-racism and equity-focused components to better capture these practices (Bottiani et al., 2025).

### Effects on Student Behavior and Discipline Disproportionality

The *R-CITY* condition demonstrated a significant effect on physical aggression, a key target for school-based violence prevention. Specifically, classrooms where teachers received *R-CITY*'s equity-focused curriculum and coaching showed significantly lower observed Physical Aggression across all students compared to classrooms receiving traditional SEL only. This finding is noteworthy as it reflects improved behavioral climate for the entire classroom. However, there were no significant effects on other observed student behaviors, such as Meaningful Participation, Cooperation, or Socially Disruptive Behavior. Verbal Aggression was significantly reduced in unadjusted analyses but did not remain significant after the B-H correction.

The reduction in Physical Aggression supports an underlying premise of this trial's design. Despite strong evidence that racism and discrimination predict youth aggression and violence, prevention efforts have typically focused on helping individuals cope with discrimination rather than preventing discriminatory experiences (National Institute on Minority Health & Health Disparities, 2017). The trial was premised on the theory that preventing exposure to racism and discrimination, rather than solely building coping skills, could reduce youth violence by addressing these experiences at their source. While building youth resilience to racial stress remains important (Anderson & Stevenson, 2019), the current findings provide initial evidence that teacher-focused bias prevention can reduce physical aggression, a key violence prevention outcome. However, whether this occurred through reduced discriminatory experiences, improved classroom climate, or other unmeasured pathways requires further investigation.

Critically, the intervention showed no effects on any of the three discipline disproportionality metrics examined (base rate of Black student suspensions, relative risk ratio, or raw differential representation). These null findings are particularly important to contextualize, as research consistently demonstrates that discipline disproportionality is more strongly related to teacher subjective judgments, implicit bias, and discretionary disciplinary practices than to objective differences in student behavior (Skiba et al., 2002). Our logic model (see Fig. S2) positioned changes in teacher mindsets (e.g., reduced Racial Discomfort) and culturally responsive practices as immediate outcomes on the ultimate trajectory of closing racial discipline gaps. The absence of disproportionality effects likely reflects insufficient intensity or duration to produce the depth and types of practice changes needed to alter teacher disciplinary decision-making. Furthermore, all metrics were whole-year counts of suspensions, and thus, are not a true “post-test” examination on this outcome. If quarterly incidents had been available, it is possible final quarter suspensions would reveal different findings.

Furthermore, reductions in discipline disproportionality likely represent longer-term, systemic outcomes requiring sustained multi-year implementation to allow time for shifts in teacher racial attitudes and implicit biases to translate into changed disciplinary practices. The single-year intervention examined here, combined with implementation challenges from COVID disruptions and sociopolitical tensions, may have been insufficient for such deeply-rooted practice changes to emerge. The reduction in Physical Aggression observed across all students demonstrates improved classroom climate for all students, improved behavioral climate alone does not address the subjective biases that drive disproportionate discipline referrals (Owens, 2022).

Positive behavior frameworks and traditional SEL interventions have been critiqued for lacking attention to bias and cultural context needed to achieve equity (Cruz & Firestone, 2024; Gregory et al., 2021). The current findings suggest that supplementing traditional SEL with limited equity-focused content (six lessons plus approximately 3 hours of coaching per teacher) implemented over one school year impacted some key outcomes, but may be insufficient to disrupt the implicit biases and subjective decision-making patterns that perpetuate discipline disproportionality. More intensive equity-focused supports specifically targeting teacher disciplinary decision-making, comprehensive transformational SEL approaches fully integrating equity, sustained multi-year implementation, or explicit training in preventing implicit bias in disciplinary encounters may be necessary to measurably close racial discipline gaps.

### Limitations

Several limitations should be considered when interpreting these findings. Measurement challenges may have attenuated observed intervention effects, particularly for student outcomes. In Cohort 1 (elementary schools), observations were conducted in regular classrooms where the same teacher delivered both *R-CITY* and typical instruction to the same students throughout the day. However, in Cohorts 2–4 (which included middle schools), students rotated between teachers and received R-CITY lessons during designated advisory periods. Although our protocol included some advisory observations, staffing constraints prevented exclusive reliance on these observations. The current analyses used morning observations as a proxy, which ensured teacher participation but meant we may have observed students who had not directly received the *R-CITY* equity-focused curriculum in advisory. More targeted measurement, such as observing only during advisory periods where intervention content was delivered, could have yielded stronger effects but was not feasible given resource constraints.

Additionally, we report here the results of intent to treat analyses, while acknowledging that the implementation fidelity of the implementation support and intervention (i.e., for both *Second Step* program and *R-CITY* equity lessons) was less than optimal. The differential findings between the full sample and Cohorts 2–4 analyses highlight the substantial impact that implementation context can have on intervention effectiveness. The variability in implementation quality across cohorts limits our ability to draw definitive conclusions about efficacy under typical conditions. Relatedly, though differential attrition could introduce bias if non-random, attrition rates were modest and similar across conditions (12% intervention, 8% comparison). Although the equity-focused coaching was integral to the intervention as designed, we acknowledge that the additional coaching dosage received by the teachers in the intervention condition represents a potential limitation, as this differential exposure may have contributed to the observed effects.

Finally, the program was implemented within the context of a *Second Step* district scale-up effort. While the *Second Step* lesson implementation fidelity also varied and was lower than expected, the schools in both conditions nonetheless implemented the program with higher fidelity (i.e., dosage) than for schools not participating in the RCT. On average, participating teachers completed more *Second Step* lessons compared to district-wide levels of implementation. Higher engagement in the *Second Step* lessons for study participants, regardless of condition, was apparent across cohorts compared to district averages of teachers not participating in the RCT (Year 1: 34%, Year 2: 26%, Year 3: 45%; Year 4: 29%; see Table S1). *Second Step* implementation was higher in Year 1 than Years 2–4, which we hypothesize was because of the elementary school composition of Cohort 1, where implementation was generally higher (relative to secondary schools) districtwide. Future studies will explore variation in outcomes by both coaching (see Franco et al., 2025) and program implementation.

## Conclusion

Taken together, this study’s findings provide initial evidence that supplementing traditional SEL with equity-focused content and coaching can produce modest but meaningful effects on teacher racial discomfort, self-efficacy, and student physical aggression, an important violence prevention target. These effects emerged despite an active comparison condition receiving extensive SEL instruction and despite substantial implementation challenges. However, the intervention did not achieve observed changes in teacher practices, including cultural responsiveness, which were a key theorized pathway. Racial disproportionality also remained unchanged. This pattern of findings raises important questions about intervention design and implementation, as well as measurement. Existing observational measures may not adequately capture the specific anti-bias and justice-oriented behaviors targeted by equity-focused interventions, suggesting the importance of new measures that incorporate these foci (Bottiani et al., 2025). Determining the conditions under which equity-focused interventions can achieve systemic practice changes will require future studies with longer implementation periods, more intensive supports, and measures designed to capture anti-bias teaching behaviors.

## Supplementary Information

Below is the link to the electronic supplementary material.

ESM 1 DOCX (346 KB)

## Data Availability

The data are available through the University of Virginia’s DATAVERSE at https://dataverse.lib.virginia.edu, doi:10.18130/V3/NVF4UG, Project Number NVF4UG.

## Declarations

### Clinical Trial Registration

See ClinicalTrials.gov number NCT05585918.
