# Supplementary material for: The R-CITY Youth Violence Preventive Intervention: Primary Outcomes from a School Cluster Randomized Controlled Trial
Source: Prev Sci. 2026 Apr 15;27(5):843–58. doi: 10.1007/s11121-026-01907-1 (PMC13421272; doi:10.1007/s11121-026-01907-1)
Supplement: Supplementary file 1 — DOCX (346 KB) [file 11121_2026_1907_MOESM1_ESM.docx]

**Randomization Procedure**

Randomization was conducted using the nbpMatching package in R (Beck et al., 2016; R Core Team, 2021) for the first three cohorts. nbpMatching was used to generate a distance matrix for select school covariates, in which each covariate was optimally weighted according to a reweighted Mahalanobis distance. Seven covariates were used in all three cohorts based on theory and priority of balance between groups: (1) prior suspension rate, (2) percent of students qualifying for free or reduced-price meals, (3) percent of students identified as economically disadvantaged, (4) racial composition of students in the school, (5) percent of students identified as being English Language Learners, (6) percent of students chronically absent, and (7) percent of students proficient on the standardized test. An eighth covariate, school type (elementary vs. middle school), was added for the second and third cohorts, which included both school types. Pairwise matches were created with a goal of minimizing the sum of the distance between pairs by conducting a total of 10,000 iterations and ensuring that each covariate resulted in a maximum distance of less than 1 across all iterations. Finally, the two groups of school pairs were randomly assigned to a dichotomous treatment group using a coin flip. Independent *t*-tests were conducted across a range of 20 covariates (i.e., the covariates used in weighting, plus other covariates of interest) to compare group means. There were no significant differences across any covariates examined, indicating a well-balanced randomized design. To ensure that balance was achieved across all cohorts, 12 pairwise matches that achieved adequate matching quality were generated for the second and third cohort. Balance across all study cohorts available was then assessed. While each set of matched pairs produced balance on the 20 key covariates within the new cohort; some matches would have perturbed balance in the full study sample, depending on the randomization outcome of the new cohort. Thus, a paired match that resulted in balance of covariates in the full sample was selected and used in the final randomization step (coin flip).

Due to the small sample size in the fourth cohort (*n* = 3 schools), the standard matching optimization procedure was not feasible and an alternative randomization approach was used. The study team determined that two schools would be assigned to the intervention condition and one to the comparison condition, yielding three possible randomization outcomes. To ensure overall sample balance, all three scenarios were evaluated across the 20 key covariates; none would have disrupted balance in the full study sample. Therefore, a random number was generated in R using the runif() function to produce a value between 0 and 3, and the ceiling of this number (an integer from 1 to 3) determined which school served as the comparison school.

**Table S1**

*Teachers’ Self-Reported Lesson Implementation*

|  | Intervention  Condition | | | | | Comparison  Condition | | |  |
| --- | --- | --- | --- | --- | --- | --- | --- | --- | --- |
| **Cohort 1** |  | *n* | *M* | *SD* | *n* | | *M* | *SD* | |
| Proportion of *R-CITY* Lessons Completed |  | 23 | .05 | .14 | - | | - | - | |
| Proportion of *Second Step* Lessons Completed |  | 21 | .80 | .31 | 10 | | .97 | .05 | |
| **Cohort 2** |  | *n* | *M* | *SD* | *n* | | *M* | *SD* | |
| Proportion of *R-CITY* Lessons Completed |  | 18 | .69 | .26 | - | | - | - | |
| Proportion of *Second Step* Lessons Completed |  | 18 | .28 | .23 | 21 | | .37 | .30 | |
| **Cohort 3** |  | *n* | *M* | *SD* | *n* | | *M* | *SD* | |
| Proportion of *R-CITY* Lessons Completed |  | 22 | .94 | .22 | - | | - | - | |
| Proportion of *Second Step* Lessons Completed |  | 19 | .54 | .30 | 20 | | .64 | .33 | |
| **Cohort 4** |  | *n* | *M* | *SD* | *n* | | *M* | *SD* | |
| Proportion of *R-CITY* Lessons Completed |  | 8 | .94 | .12 | - | | - | - | |
| Proportion of *Second Step* Lessons Completed |  | 9 | .41 | .25 | 5 | | .18 | .17 | |


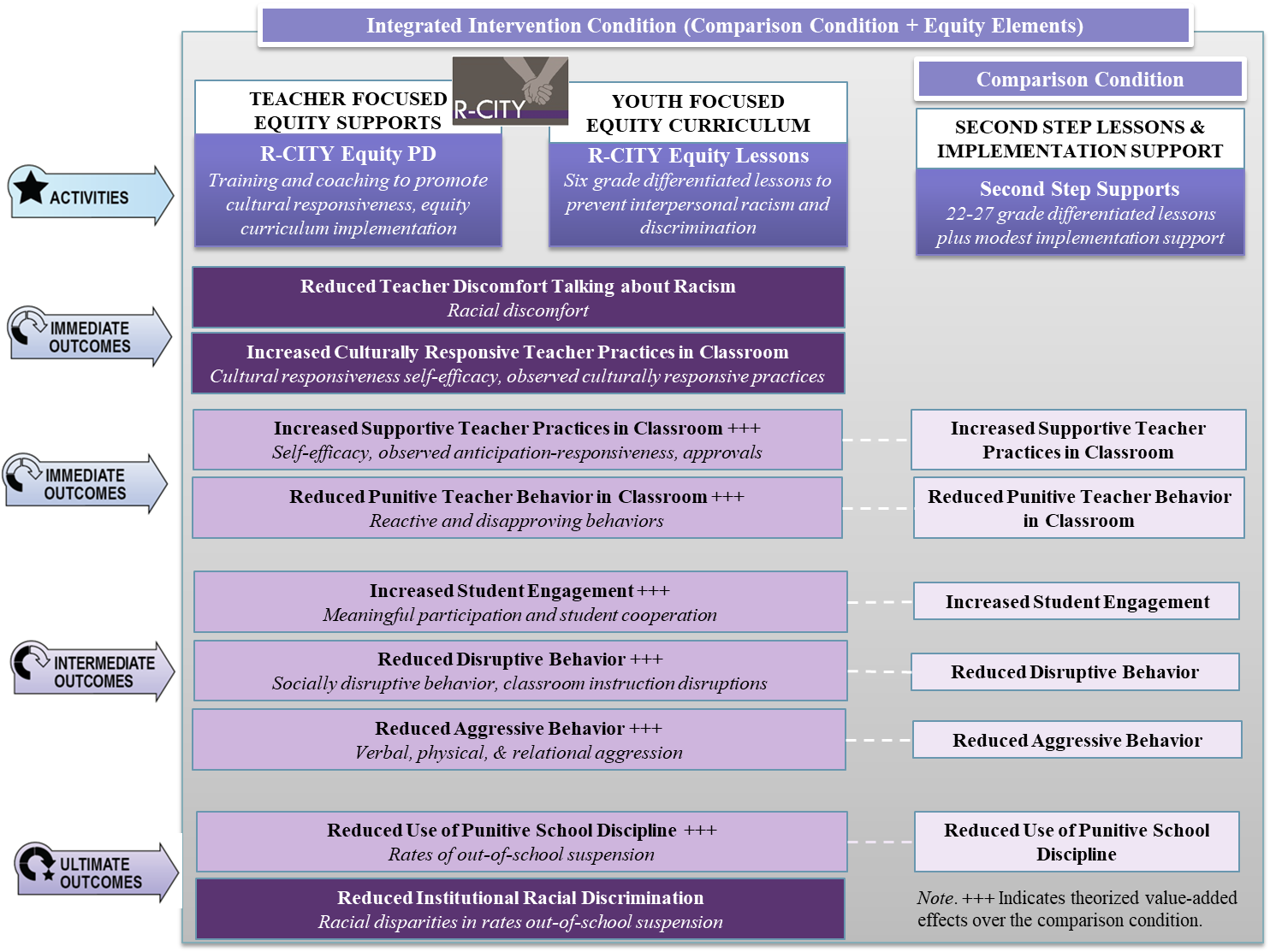


*Figure S1*. R-CITY Logic Model

*Figure S2***.** CONSORT Figure for the R-CITY School Cluster Randomized Trial

## Recruitment

Excluded (*n* = 976)

Ineligible via inclusion criteria (*n* = 599)

Declined to participate
(*n* = 377)

Declined to participate (*n* = 278)

Assessed for eligibility
*N* = 1,121 teachers

## Allocation

Analyzed for primary outcomes (*n* = 72 teachers)

Analyzed for primary outcomes (*n* = 73 teachers)

**Comparison Condition**

*Completed post data collection:*

Teacher survey (*n* = 62)

Classroom observations (*n* = 65)

**Intervention Condition**

*Completed post data collection:*

Teacher survey (*n* = 58)

Classroom observations (*n* = 59)

Allocated to **Comparison**
 *j* = 13 schools; *n* = 72 teachers

*Completed baseline data collection:*

Teacher survey (*n* = 66)

Classroom observations (*n* = 71)

*Engaged in SEL Only Coaching (n = 71)*

Allocated to **Intervention**
*j* = 14 schools; *n* = 73 teachers

*Completed baseline data collection:*

Teacher survey (*n* = 71)

Classroom observations (*n* = 73)

Engaged in SEL + Equity Coaching (n = 70)

## Follow Up

## Analysis

## Enrollment

Randomized
*J* = 27 schools
*N* = 145 teachers

**Table S2**. Baseline equivalence tests comparing teachers with complete data (pre & post) to those with pre-test data only

| **Teacher Demographics** | **Pre & Post**  **(n = 118)** | | **Pre-Only**  **(n=21)** | | **Baseline Equivalence** |
| --- | --- | --- | --- | --- | --- |
|  | **n** | **%** | **n** | **%** |  |
| Teacher of Color | 19 | 16.10 | 3 | 14.29 | *X*^2^(1) < .001,  *p* > 0.999 |
| Woman | 92 | 79.31 | 18 | 85.7 | *X*^2^(1) = 0.145,  *p* = 0.703 |
| STEM Subject | 32 | 27.33 | 7 | 33.33 | *X*^2^(1) = 0.103,  *p* = 0.749 |
| Middle School | 54 | 46.15 | 9 | 42.86 | *X*^2^(1) = 0.002,  *p* = 0.967 |
|  | **M** | **SD** | **M** | **SD** |  |
| Years Teaching | 11.30 | 8.24 | 10.07 | 6.19 | *t*(34.036) = 0.793,  *p* = 0.43 |
